# Bilateral renal hemorrhage in an anticoagulated patient: A rare case of Wunderlich syndrome

**DOI:** 10.1016/j.radcr.2024.04.009

**Published:** 2024-04-24

**Authors:** Federica Masino, Manuela Montatore, Annalori Panunzio, Rossella Gifuni, Domenico Mannatrizio, Marina Balbino, Gianmichele Muscatella, Giuseppe Guglielmi

**Affiliations:** a Department of Clinical and Experimental Medicine, Foggia University School of Medicine, Viale L. Pinto 1, 71121, Foggia FG, Italy; b Radiology Unit, “Dimiccoli” Hospital, Viale Ippocrate 15, 70051, Barletta BT, Italy; c Radiology Unit, “IRCCS Casa Sollievo della Sofferenza” Hospital, Viale Cappuccini 1, 71013 San Giovanni Rotondo FG, Italy

**Keywords:** Hemorrhage, Wunderlich syndrome, WS, Renal hemorrhage, Emergency, CT

## Abstract

We describe a rare case of Wunderlich syndrome with bilateral renal hemorrhage in a patient under anticoagulant therapy for atrial fibrillation. An 84-year-old woman came to our department complaining of acute bilateral flank pain. Clinical and laboratory examinations revealed a condition of hypovolemic shock. An abdominal contrast-enhanced CT scan detected the presence of a bilateral hemorrhage affecting the peri- and para-renal spaces. Planning an appropriate management strategy considering the anticoagulated treatment required a multidisciplinary approach in the case of the Wunderlich syndrome diagnosis.

## Introduction

Wunderlich syndrome (WS) is an uncommon and potentially fatal condition characterized by non-traumatic renal bleeding into the subcapsular, perirenal, and/or pararenal areas. Carl Wunderlich is credited with making the first clinical description of WS in 1856 [1]. Patients may present a wide range of symptoms, ranging from vague stomach or flank pain to more severe conditions like hypovolemic shock. A tiny percentage of individuals experience the traditional Lenk triad of symptoms, which includes flank pain, a flank mass, and hypovolemic shock [2]. The etiology, which must be non-traumatic, can be classified as neoplastic and non-neoplastic. The majority of WS cases (60%-65%) are caused by neoplasms, which include malignancies such as renal cell carcinoma as well as benign neoplasms, the most prevalent of which is angiomyolipoma. Aout 30%-35% of all cases of WS have non-neoplastic causes, which include vascular conditions such as vasculitis syndromes (polyarteritis nodosa being the most common), renal artery aneurysms or pseudoaneurysms, arteriovenous malformations, and fistulas, renal vein thrombosis, cystic renal diseases, calculus disease, nephritis, and coagulation disorders. The remaining 10% can be attributed to idiopathic causes [1,2]. Patients with a history of urinary tract infections, particularly pyelonephritis, renal cystic disease, severe infections, diabetes, hypertension, end-stage renal disease, and iatrogenic causes (systemic anticoagulation) are at greater risk of developing WS [3].

The imaging technique for diagnosing WS is contrast-enhanced CT (CECT). It makes it possible to identify renal hemorrhage, identify its exact location and extension, characterize its underlying causes, and make treatment management easier [4].

## Case presentation

### Anamnesis

An 84-year-old woman came to our department complaining of acute bilateral flank discomfort that was getting worse and spreading throughout her abdomen. The pain was more noticeable on the left side. She was pale, sweating, and cyanotic. She had a history of persistent atrial fibrillation and was on anticoagulants. She denied experiencing any trauma.

### Diagnostic evaluation

**Fig. 1:**
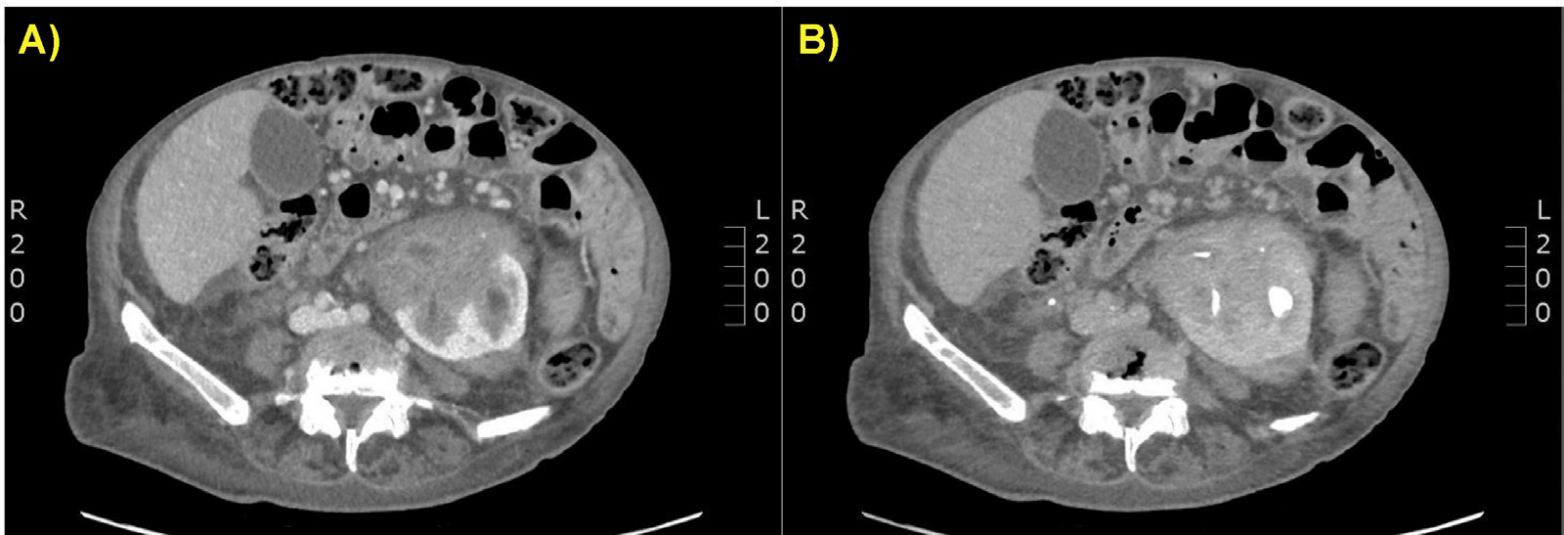
(A and B) Contrast-enhanced CT scan on axial planes. Venous (A) and delayed (B) phases showed altered morphology of the left kidney, largely occupied by an inhomogeneous hilar, and parenchymal renal area associated with a peri-renal and para-renal fluid effusion.

**Fig. 2:**
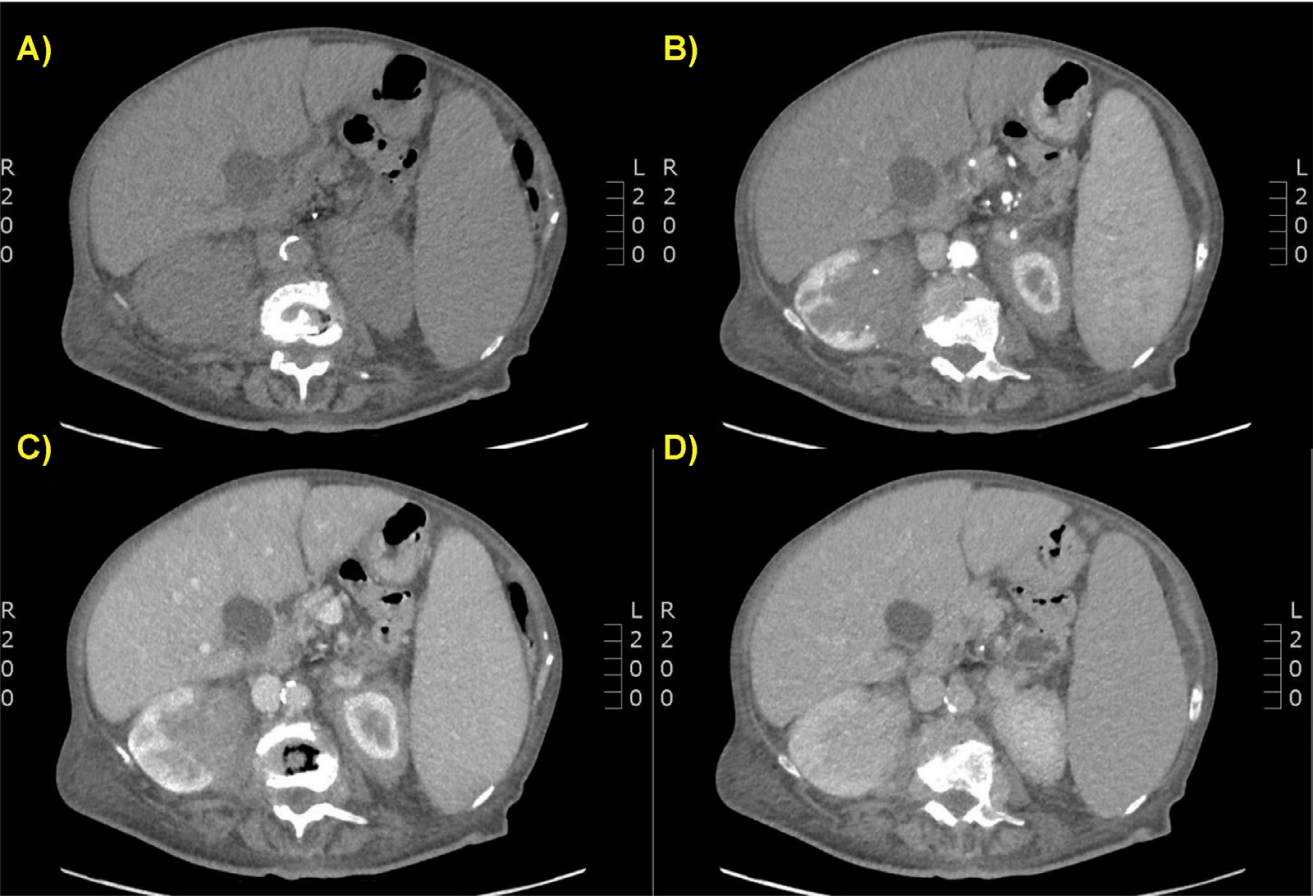
(A-D) Contrast-enhanced CT scan on axial planes. Non-contrast (A), arterial (B), venous (C), and delayed (D) phases showed altered morphology of the left kidney, partially occupied by an inhomogeneous hilar, and parenchymal renal solid area, associated with peri-renal and para-renal fluid effusion.

**Fig. 3:**
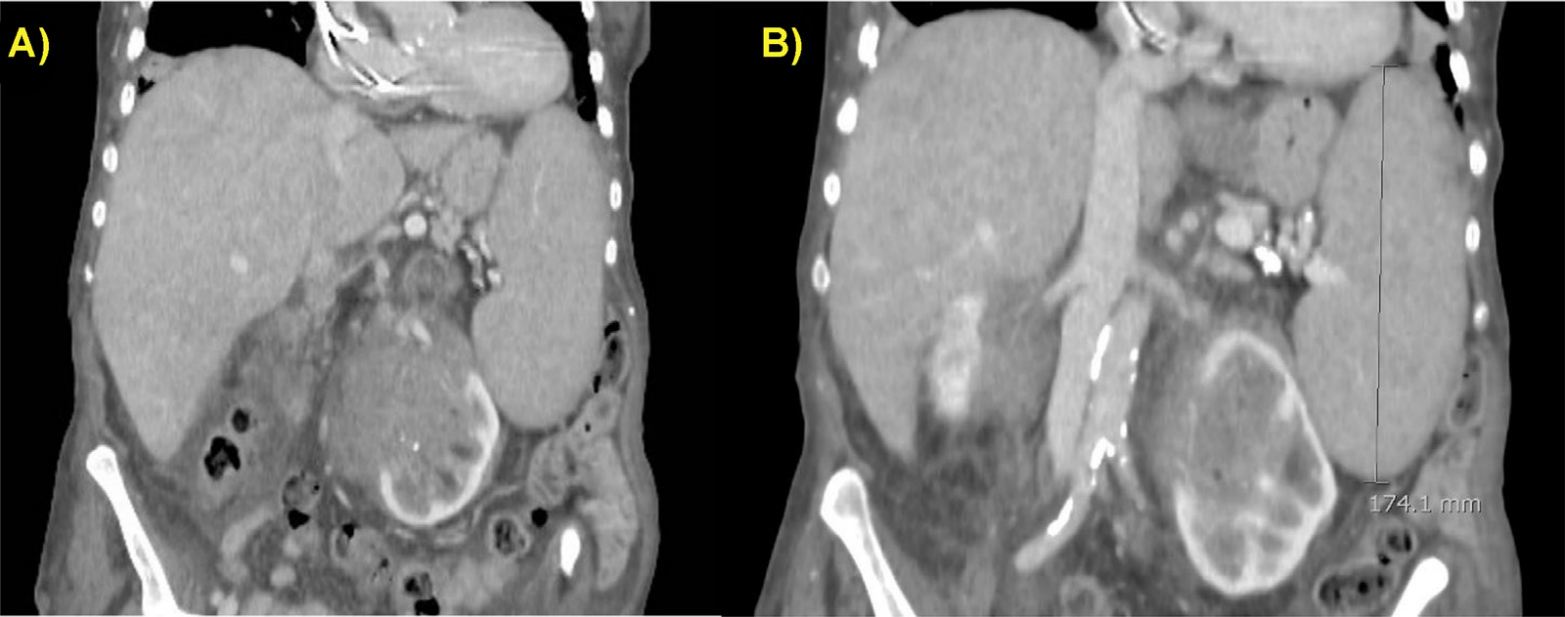
(A and B) Contrast-enhanced CT scan on coronal planes showed the left kidney hemorrhage (A) with concomitant splenomegaly (maximum diameter of 17 cm) and liver enlargement.

The patient presented tachycardia and hypotension. Her hemoglobin level in the blood was 7 g/dL and urine analysis revealed hematuria. The creatinine level was 0.9 mg/dL. A condition of hypovolemic shock was diagnosed, and fluid infusion was administered. An abdominal CECT was requested; it was performed a standard protocol for multiphasic renal CT acquisition with a non-contrast phase followed by a contrast-enhanced acquisition in the corticomedullary (40-70 seconds), nephrographic (80-100 seconds), and excretory (3- and 5-minute delay) phases. Maximum intensity projection and multiplanar reformation images of multiphasic CT were performed. A great part of the left renal parenchyma and hilar region was occupied by a large inhomogeneous, solid area, with a maximum diameter of 9 cm, with a densitometry also with a partially hematic component, extending into the pararenal space. On the left, after medium contrast administration there was increased densitometry as per signs of weak active supply. Homolateral renal pelvis and calyceal groups were poorly identifiable. Similar findings were appreciated in the right kidney of smaller size and maximum diameter of 7 cm. There was perihepatic and peri-splenic fluid effusion. No malignant tumors or kidney masses were detected (Fig. 1, Fig. 2, Fig. 3).

As a result of the clinical and imaging correlation, a diagnosis of WS was made. The use of anticoagulants was discontinued, and transfusions of blood were carried out. Her postblood transfusion hemodynamic stability led to conservative management, including bed rest and close observation. Anticoagulant therapy was resumed once the hematoma was almost completely reabsorbed, as revealed by the ultrasound monitoring examinations carried out in a short period of time. The patient was discharged in good clinical condition when hemoglobin and renal function returned to normal.

## Discussion

This case report showed a rare occurrence of WS with bilateral kidney involvement. The patient was following an anticoagulant treatment, which is not considered as a common cause of WS. Moreover, anticoagulant therapy may present urologic complications such as hematuria but rarely spontaneous hemorrhage with perirenal and/or pararenal hematoma [1,5].

Imaging plays a crucial role in a suspected case of WS as it confirms the diagnosis by detecting the renal hemorrhage; gives information on the extent and location of the bleeding in the subcapsular, perirenal and/or pararenal space; and provides a possible underlying cause for the bleeding, since it might show renal vascular structure, origins of tumors and pathological change in adjacent tissues [2,6]. CT has the highest sensitivity in identifying perirenal hemorrhage among the imaging modalities. For this reason, abdomen CT is often the initial modality of choice and is pivotal to the diagnosis and management of WS [3,4]. To detect the presence of blood in CT, it is necessary to consider the various densities it assumes depending on the time of hemorrhage. For instance, acute hemorrhage shows a density of between 40 and 70 Hounsfield units (HU) in the non-contrast phase, while clotted blood has a higher density, closer to 60 HU. Blood shows lower attenuation values if the image is taken 48 hours after the bleeding event. In the case of hemoperitoneum, it is possible to detect the so-called sentinel clot, which shows clots close to the bleeding site. This sign is difficult to identify in the case of retroperitoneal hemorrhage, and it could not be identified in the reported case. After contrast medium administration, on the venous phase, the hemorrhage is hypodense to iso-dense in relation to the enhanced renal parenchyma [7]. Post-contrast scans are critical to assess the presence of an active extravasation of contrast material indicative of ongoing hemorrhage requiring urgent consultation [2,6].

**Fig. 4:**
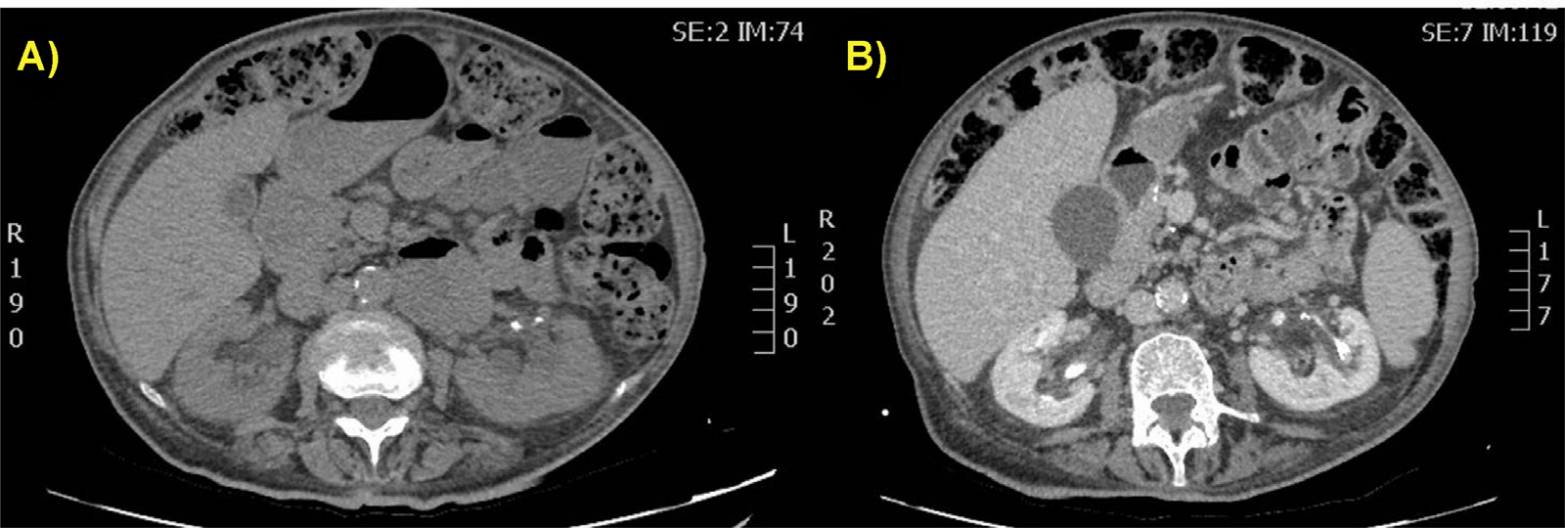
(A and B) Contrast-enhanced CT scan performed 6 months on the same patient before the bilateral renal hemorrhage occurred. Non-contrast (A) and venous (B) phases on axial planes showed regular morphology, dimension, and density of both kidneys in absence of expansive lesions. Left kidneys presented some calyceal calcifications.

It is not always possible to identify the cause of bleeding on CT examination. In half of the cases, a ruptured aneurysm or angiomyolipoma, for example, is not recognizable because the hematoma itself, being large, obscures the underlying cause. In these cases it becomes necessary to perform a new CT scan 8 weeks after the initial examination [1]. In the presented case, it was useful to compare the examination with a previous similar CT examination performed 6 months earlier, in which no kidney anomalies or vascular anomalies were detected. It was therefore possible to exclude the underlying presence of renal masses more easily, which also rarely occur bilaterally (Fig. 4).

The patient's anticoagulant therapy was identified as the cause of the bleeding. Currently, to our knowledge, similar cases of WS associated with anticoagulant therapy have rarely been described [3,6]. The management of WS can be difficult, especially in anticoagulated patients since there are no well-defined clinical guidelines for management. The management of WS essentially depends on the presence of active bleeding and the patient's hemodynamic status. Patients with massive hemorrhages must be stabilized promptly with conservative measures and if necessary with angiographic or surgical treatments [7,8]. Therapeutic options include conservative management with angiotensin receptor blockades, early volume resuscitation with intravenous fluids, and blood transfusion [3,5]. When imaging detects active extravasation, the preferred approach is angiography and embolization, while partial or radical nephrectomy is reserved for neoplastic causes or refractory bleeding [2,9]. Since in the reported case the patient was taking anticoagulant medication, conservative treatment was considered the most appropriate and turned out to be an effective and optimal action strategy. Furthermore, the extravasations stopped spontaneously, and the hematoma reabsorbed in a few weeks. The patient was kept under observation and monitored with renal ultrasound scans performed at short times. They showed a progressive reduction in the size of the hematoma and confirmed the absence of renal pathologies susceptible to surgical intervention. When the hematoma was nearly completely healed and it was found that there was no renal pathology that required surgical treatment, the anticoagulant therapy was resumed. When renal function and hemoglobin level were stable, the patient was discharged in good clinical condition.

## Conclusion

Wunderlich syndrome (WS) is an uncommon and possibly fatal condition characterized by non-traumatic renal hemorrhage in the subcapsular, perirenal, and/or pararenal spaces. To improve the management of WS patients, clinicians should be aware of this potentially fatal illness while approaching patients who report flank discomfort and are on anticoagulant treatment. It is crucial to increase awareness of this disease among healthcare professionals in order to enable prompt diagnosis and treatment, hence enhancing patient outcomes and quality of life.

## Author's contribution

All Authors contributed to the final manuscript.

## Patient consent

Complete written informed consent was obtained from the patient for the publication of this study and accompanying images.
